# Zebra Alphaherpesviruses (EHV-1 and EHV-9): Genetic Diversity, Latency and Co-Infections

**DOI:** 10.3390/v8090262

**Published:** 2016-09-20

**Authors:** Azza Abdelgawad, Armando Damiani, Simon Y. W. Ho, Günter Strauss, Claudia A. Szentiks, Marion L. East, Nikolaus Osterrieder, Alex D. Greenwood

**Affiliations:** 1 Leibniz-Institute for Zoo and Wildlife Research, Alfred-Kowalke-Strasse 17, Berlin 10315, Germany; azza@izw-berlin.de (A.A.); szentiks@izw-berlin.de (C.A.S.); EAST@izw-berlin.de (M.L.E.); 2 Institut für Virologie, Freie Universität Berlin, Robert-von-Ostertag-Str. 7-13, Berlin 14163, Germany; adamiani@zedat.fu-berlin.de (A.D.); no.34@fu-berlin.de (N.O.); 3 School of Life and Environmental Sciences, University of Sydney, Sydney, NSW 2006, Australia; simon.ho@sydney.edu.au; 4 Tierpark Berlin-Friedrichsfelde, Am Tierpark 125, Berlin 10307, Germany; g.strauss@tierpark-berlin.de; 5 Department of Veterinary Medicine, Freie Universität Berlin, Oertzenweg 19, Berlin 14163, Germany

**Keywords:** EHV-1, EHV-9, zebra, latency, diversity, co-occurrence

## Abstract

Alphaherpesviruses are highly prevalent in equine populations and co-infections with more than one of these viruses’ strains frequently diagnosed. Lytic replication and latency with subsequent reactivation, along with new episodes of disease, can be influenced by genetic diversity generated by spontaneous mutation and recombination. Latency enhances virus survival by providing an epidemiological strategy for long-term maintenance of divergent strains in animal populations. The alphaherpesviruses equine herpesvirus 1 (EHV-1) and 9 (EHV-9) have recently been shown to cross species barriers, including a recombinant EHV-1 observed in fatal infections of a polar bear and Asian rhinoceros. Little is known about the latency and genetic diversity of EHV-1 and EHV-9, especially among zoo and wild equids. Here, we report evidence of limited genetic diversity in EHV-9 in zebras, whereas there is substantial genetic variability in EHV-1. We demonstrate that zebras can be lytically and latently infected with both viruses concurrently. Such a co-occurrence of infection in zebras suggests that even relatively slow-evolving viruses such as equine herpesviruses have the potential to diversify rapidly by recombination. This has potential consequences for the diagnosis of these viruses and their management in wild and captive equid populations.

## 1. Introduction

Alphaherpesviruses are an ancient group of double-stranded DNA viruses that diverged from other herpesviruses ~200 million years ago [1]. Novel alphaherpesviruses are frequently discovered, and the extent of their genetic diversity is unknown. Differences in virulence and in biological and biochemical properties form an important basis for distinguishing between related herpesviruses, different virus strains and isolates [1]. Equine herpesviruses 1 (EHV-1) and 9 (EHV-9) are classified within the genus *Varicellovirus*, subfamily Alphaherpesvirinae, in the Herpesviridae family of the order Herpesvirales [2,3]. EHV-1 and EHV-9 are unusual among herpesviruses, which are generally considered species-specific, due to their ability to infect species other than their natural hosts [4,5]. Infections of non-definitive hosts can cause histopathological changes ranging from non-suppurative encephalitis to neuronal degeneration. Viral DNA has been detected in cases with high fatality rates in these hosts [4,5,6,7].

We recently conducted a large-scale seroprevalence survey using a discriminatory specific peptide-based enzyme-linked immunosorbent assay (ELISA), with the aim of determining the host range and the possible reservoirs for EHV-1 and EHV-9 among zoo and wild animals. EHV-1 and EHV-9 exhibit a broad host range across mammalian families, including members of the Equidae, Rhinocerotidae and Bovidae, with a high prevalence of EHV-1 and EHV-9 antibodies in different geographic areas and habitats [8]. Serological analysis was undertaken to avoid the underestimation of prevalence by relying on viral antigen or nucleic acid and, thus, the study did not provide information about the general genetic diversity of the circulation of EHVs in zebra populations. Little is known about the genetic characterization and diversity of EHV-1 and EHV-9 isolates circulating in zoo animals and wildlife. Studies suggest that zebras are the natural host for EHV-9, although serological prevalence also points to African rhinoceroses as an additional potential natural host [4,8,9,10].

The full genome of a single isolate of a zebra-borne EHV-1 strain has been sequenced from Grevy’s zebra [11]. Less genetic information is available for EHV-9 [3,4,6,7,10]. Therefore, the extent of strain divergence in wild and captive zebra populations is unknown. Considering that novel divergent EHVs have been associated with disease and fatalities among zoological collections, determining the genetic diversity and potential virulence of different strains is critical to the management of wild-derived equids in such collections.

Viruses from the subfamily *Alphaherpesvirinae*, including EHV-1, have the ability to establish a lifelong latent state in neurons of the sensory ganglia or in lymphoid cells, likely cells of the monocytic and T-cell lineages in the case of EHV-1 [12,13,14]. The expression of lytic viral genes is repressed in latency and only a limited portion of the genome, usually comprising the so-called latency-associated transcripts (LATs), is expressed during the quiescent phase of infection [15]. Latency-associated transcripts have been shown to be transcribed from the DNA strand opposite to that encoding open reading frame 63 (ORF63) mRNA, the *infected cell polypeptide 0* (*ICP0*) homolog [13,15,16,17]. The ability of herpesviruses to establish lytic and subsequent latent infection is an important epidemiological advantage for the virus to evade the host immune system during the latent phase and to establish a persistent infection [18,19]. There is indirect evidence of EHV-9 latent infection in the trigeminal ganglion of free-living Burchell’s zebras [10].

Recombination and reassortment are mechanisms that are widely adopted by RNA viruses to increase their diversity. This is particularly pronounced in influenza viruses and retroviruses [20]. Genetic recombination has also been reported among alphaherpesviruses of high genetic similarity when infecting the same horse host at the same time [1,21]. Natural recombination in the *ICP4* gene between different virus species such as EHV-1 and EHV-4 has been observed in horses [22]. A recent study of EHV-1 and EHV-4 isolates from horses in Australia and New Zealand demonstrated widespread recombination between EHV-1 and EHV-4, in particular [23]. Among EHVs isolated from wild equids, infection with a recombinant zebra-borne EHV-1/EHV-9 strain was reported in a polar bear and in an Indian rhinoceros [24,25]. Whether recombination occurred in zebras in Africa or zoos elsewhere is unclear. However, viral co-infection is necessary for interspecific recombination in herpesviruses [1].

Here, we report both lytic and latent EHV-9 and EHV-1 infections in zoo and wild zebras and evaluate the relative genetic diversity in these viruses. Our study demonstrates, for the first time, an incidence of co-infection of EHV-9 and EHV-1 in a zebra. Our results collectively demonstrate that zebras can serve as a natural host for both viruses, with their co-occurrence suggesting an origin for recombinant zebra EHV strains.

## 2. Materials and Methods

### 2.1. Sample Collection

Samples were obtained from 17 zoo equid animals: 14 animals necropsied at the Leibniz Institute for Zoo and Wildlife Research (Berlin, Germany) between 2012 and 2015 (three plains zebras (*Equus quagga boehmi)*, three Grevy’s zebras (*Equus grevyi)*, two Hartmann’s mountain zebras (*Equus zebra hartmannae)*, three Somali wild asses (*Equus africanus somalicus)*, and three donkeys (*Equus africanus asinus*), two animals from Zoo Hannover (Germany) (Somali wild asses) and one animal from Réserve Africaine de Sigean (France) (Somali wild ass) (Table S1). Tissues were collected from different organs, focusing on respiratory and nervous tissues. Respiratory lymph nodes, superior tracheobronchial, paratracheal and pulmonary lymph nodes, and both left and right trigeminal ganglia were collected from ten animals to investigate viral latency. Sampling of trigeminal ganglia and lymph nodes was not possible from the other seven animals. In addition, brain tissues were opportunistically collected from carcasses of seven free-ranging plains zebras (*Equus quagga)* (Tanzania) killed by predators. The samples were stored immediately in RNAlater (Thermo Fischer Scientific, Vilnius, Lithuania) at −80 °C.

### 2.2. Histopathology

For histology investigations, specimens from different organs, lung, liver, heart, spleen, lymph nodes, olfactory bulb, and brain were collected and fixed in 4% neutral buffered formalin. Fixed tissue samples were routinely embedded in paraffin wax post-necropsy and serially sectioned at 4 µm and stained with hematoxylin–eosin (HE).

### 2.3. DNA and RNA Extraction

Viral DNA was extracted from tissues, nasal epithelium, lung, liver, heart, spleen, lymph nodes, olfactory bulb, and brain, using the QIAamp DNA Mini Kit (Qiagen, Hilden, Germany) according to the manufacturer’s instructions [26]. Total RNA was extracted from lymph nodes and trigeminal ganglia using an RNeasy Plus Mini Kit (Qiagen). For elimination of genomic DNA (gDNA), RNA was processed with the RQ1 RNase-Free DNase kit (Promega, Madison, WI, USA) according to the manufacturer’s protocol. Before complementary DNA (cDNA) synthesis, RNA extraction from all samples was tested for gDNA absence using the housekeeping gene β-glucuronidase as a marker. Samples that were gDNA-free were further processed for cDNA synthesis, while positive samples were re-treated with RNase-Free DNase kit and re-tested. To avoid the risk of virus underestimation after sample treatment, the RNA concentration was measured after testing for DNA background. Subsequently, 1 μg of RNA was reverse-transcribed to cDNA using Superscript III (Thermo Fischer Scientific, Carlsbad, CA, USA) and either random hexamer primers [27] or an LAT-specific primer, designed for first time in this study to generate a specific LAT-cDNA strand, for *gB* and LAT, respectively. Precautions were taken to avoid laboratory contamination during sample processing, including the use of disposable labware and separate facilities for DNA, RNA isolation and PCR amplification. Positive EHV-1 DNA, DNA-free and RNA-free water, and RNA and DNA extracted from swabs from the work areas (negative control (NC) swabs) were used as positive and negative controls.

### 2.4. Molecular Characterization

PCR reactions using MyTaq HS polymerase mix (Bioline, Luckenwalde, Germany), 200 nM primers, and approximately 200 ng of the extracted template DNA were performed [24]. Screening of herpesviruses using nested PCR targeting a partial sequence of the DNA-dependent DNA polymerase gene (*Pol*, *UL30*) was performed as described previously [28]. Amplification and sequencing of the *Pol*, *gB*, *UL49.5*, *ORF15* and genes encoded in the unique short segment (*ORF69*–*ORF74*; protein kinase, *gG*, *gp2*, *gD*, *gI*, and *gE*) was performed using primers specific to EHV-1 and EHV-9. The amplified products were purified and directly sequenced. The primers for nested PCR amplification, *ORF15* and *UL49.5* have been described previously [24]. New primers used in this study are listed in Table S2. The nucleotide sequences obtained in this study have been deposited in GenBank (accession numbers: KX101085–KX101111).

### 2.5. Quantitative Real-Time PCR

For detection of latent infection in the collected lymph nodes and trigeminal ganglia, all samples were analyzed by quantitative real-time PCR (qPCR) with the Applied Biosystems 7500 FAST (ABI, Foster City, CA, USA) to detect the presence of late *gB* gene and LATs based on ORF63. Primers and probes targeting a 90 bp product of *gB* were used as previously described [13]. Specific primers and probes were designed to target 91 bp of ORF63 (Genscript, Piscataway, NJ, USA); cDNA using specific primer for LAT RNA was used as a template. The TaqMan probe (Genscript) was labeled with 6-carboxyfluorescein (6-FAM) and the quencher carboxytetramethylrhodamine (TAMRA) at the 5′ and 3′ ends, respectively. The primers shared 100% similarity with EHV-1 and 96%–100% similarity with EHV-9.

The qPCR protocol included an initial 95 °C step for 3 min, followed by 40 cycles of 95 °C for 10 s and 60 °C for 30 s. The qPCR reaction was performed in 20 μL using 1× SensiFAST™ Probe Lo-ROX Kit (Bioline, Luckenwalde, Germany), 450 nM of each primer, 100 μM of the respective TaqMan probe and 5 μL of the template. Standard curves created in this study were quantified by measuring absorbance at 260/280 nm using Nanodrop v3.5 (NanoDrop Technologies, Wilmington, DE, USA) and an online application for calculating DNA copy number (http://endmemo.com/bio/dnacopynum.php). Amplification efficiency was calculated from the slope of a standard curve generated from 10-fold dilutions of EHV-1 genomic DNA for *gB* [29] and synthetic oligonucleotides of the targeted region of ORF63 (Figure S1). Assay sensitivity was assessed as described previously [30]. DNA- and RNA-free water and NC swabs were used as negative controls with each run. DNA and mRNA quantification for the *gB* and LATs, respectively, were compared with standard curves generated for each gene. The data were then normalized to a standard curve generated with oligonucleotides specific to equine *beta-2-microglobulin* *(B2M)*. Viral DNA concentration was expressed as copies per million cells, considering that each diploid eukaryotic cell has two copies of the *B2M* gene [13,31]. For absolute quantification of the LAT, the raw LAT cDNA data were normalized to the standard curve and then normalized to the calculated cell number based on the *B2M* gene data.

### 2.6. Genetic Diversity and Phylogenetic Analyses

To estimate the mean nucleotide diversity (π), along with the average number of nucleotide substitutions per site within the EHV-1 and EHV-9 sequence groups, we used the maximum composite likelihood model in MEGA 7.0 with a gamma distribution for rate variation among sites was conducted [32,33]. Nucleotide substitutions per site between sequences were corrected using the Tamura–Nei model, while the standard error (SE) was estimated with 500 bootstrap replicates [34].

To infer evolutionary relationships, we performed phylogenetic analyses of the nucleotide sequences of *gB*, *Pol* and *UL49.5* genes isolated from zebra tissues. Reference sequences for the same regions of EHV-1, EHV-9, and EHV-4 were obtained from GenBank and aligned using ClustalW implemented in Bioedit v7.1.11 [35]. Phylogenetic trees were inferred using maximum-likelihood in RAxML v8.2.4 [36] and Bayesian inference in MrBayes v3.2.2 [37]. In the maximum-likelihood analyses, we performed a rapid bootstrapping analysis followed by a thorough search for the best-scoring tree. All trees were rooted with the EHV-4 sequences. In the Bayesian phylogenetic analysis, the GTR + G substitution model was used and a flat tree prior was specified. The posterior distributions of parameters, including the tree, were estimated using Markov chain Monte Carlo sampling. Samples were drawn every 1000 steps from a total of 10,000,000 steps. All analyses were run with one cold and one heated chain. The first 10% of samples were discarded as burn-in.

## 3. Results

### 3.1. Detection of Herpesvirus Infection

DNA was extracted from tissue samples collected from wild and zoo zebras as well as captive donkeys and Somali wild asses and investigated for the presence of equine herpesviruses using a panherpes nested PCR. EHV-9 DNA was amplified from the nasal epithelium and/or olfactory bulb of two captive Grevy’s zebras (CG2 and CG3) and brain tissue of one wild plains zebra (WP1). EHV-1 DNA was detected in the tissues of nasal epithelium and olfactory bulb of one captive plains zebra (CP4). Sequencing of the 250 bp fragment obtained from the nested PCR revealed 99% identity to previously reported sequences from the DNA polymerase (*Pol*) gene of EHV-1 and EHV-9. All tested wild asses and donkeys were negative for EHV-1 and EHV-9. Sample abbreviations and infectious status of the tested samples are shown in Table 1.

PCR assays specific to EHV-1 and EHV-9 were next applied to target complete coding sequences of *gB* (2643 bp), *Pol* (3660 bp) and *UL49.5* (400 bp; 300 *UL49.5* and 140 *UL50*) genes. In addition, partial coding sequences of *ORF15* and 9 kbp of the unique short region (*ORF69*–*ORF74*) were amplified from the positive wild and captive zebras. BLAST analysis, followed by further alignment of the obtained sequences with different EHV-1 and EHV-9 sequences from NCBI, revealed that WP1, CG2, and CG3 were infected with EHV-9, which showed high similarity to EHV-9 isolates detected in gazelle and giraffe. The 663 bp, 855 bp, and 411 bp of *gB*, *Pol*, and *UL49.5* genes, respectively, obtained from CP4 shared 99% similarity with EHV-1 isolates from horses.

### 3.2. Pathological Findings

**Figure 1:**
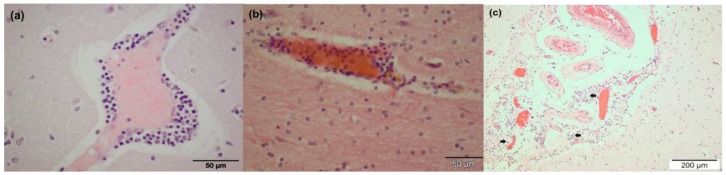
Histopathology (Grevy’s zebra, CG3) showing non-suppurative encephalitis represented by mononuclear perivascular cuffs in the gray matter of the cerebral cortex (**a**) and forebrain (**b**); Hematoxilin–eosine (HE) staining. Meningeal perivascular infiltration of inflammatory cells (arrows) (**c**); HE staining.

**Figure 2:**
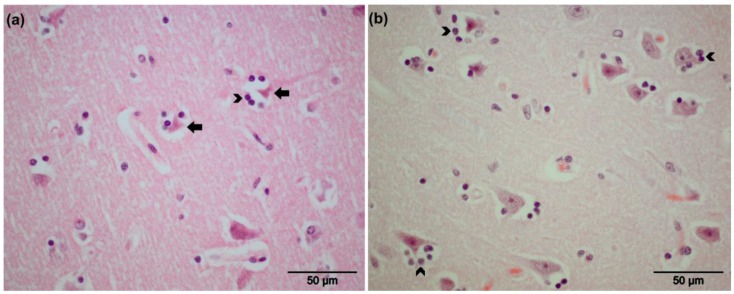
Histopathology of cerebral cortex (Grevy’s zebra, CG3) showing degenerated neurons (arrows) (**a**) with mild glial reactions (head of arrows) in (**a**,**b**); HE staining.

Gross lesions in respiratory or nervous tissue were not observed at necropsy. The clinical observations for the EHV-positive animals are described in Table 1. The captive zebra (CP4) exhibited ataxia and tremors without improvement after a course of medication. Microscopic examination revealed diffuse alveolar edema in lung tissue. The EHV-9-positive zebra (CG3) exhibited central nervous disorders with ataxia and restlessness; the animal collapsed and was subsequently euthanized. Non-suppurative encephalitis associated with perivascular cuffing in the internal granular layer (IV) of the frontal part of the cerebral cortex (forebrain) and gray matter of the cerebral cortex was observed (Figure 1a,b). Meningeal perivascular inflammation with mainly lymphocytes and macrophages was also observed (Figure 1c). Mild neuronal degeneration and glial reactions were found in the central nervous system, mainly in the cerebrum and olfactory bulb, but not in the cerebellum (Figure 2a,b). Intra-nuclear inclusion bodies were not detected. Additionally, over-dilatation of lung alveoli with congestion in intra-alveolar blood vessels was observed.

### 3.3. Detection of Latent Infection

To test for latency, DNA and RNA were extracted from respiratory lymph nodes and trigeminal ganglia. The qPCR was applied to target the late *gB* gene using DNA samples that were extracted from lymph nodes and trigeminal ganglia as a template. Out of 10 animals tested, three DNA samples obtained from trigeminal ganglia of two captive plains (CP4, CP5) and one Grevy’s (CG6) zebras tested positive for the presence of the *gB* gene. The *gB* DNA load ranged from 8 to 65.3 gene copies per million cells (Table 2). Since the identification of the virus species and individual strains using the short qPCR product is equivocal, partial coding sequences of both *gB* and *Pol* were amplified by conventional PCR. The results showed that the two zebras (CP4 and CG6) and the third zebra (CP5) were positive for EHV-9 and zebra-borne EHV-1, respectively. CG2 and CG3 tested negative for the ganglia (representing lytic infections) and thus could not be included in the latency analysis. CP4, in contrast, had both a lytic (EHV-1) and latent (EHV-9) infection and thus was included in both analyses.

To determine whether the viral DNA detected in the trigeminal ganglia was in a latent state, cDNA was tested for the presence of mRNA transcripts for *gB*. Only RNA extractions that tested negative for genomic DNA contamination using β-glucuronidase control PCRs had been transcribed to cDNA and used. In the latent state, the virus is represented by the presence of the viral genome (*gB* DNA), absence of active *gB* expression (represented by absence of mRNA), and the active expression of LATs [13]. EHV-1 and EHV-9 *gB* DNA was detected in all positive samples (CP4, CG6, and CP5), with an absence of active *gB* transcripts (Table 2). A specific LAT primer was designed and used to guarantee that only LAT RNA, and not ORF63 mRNA, was reverse transcribed to cDNA, which was used later as a template in qPCR. Expression of LATs was determined by using a specific TaqMan probe. All positive samples (CP4, CG6, and CP5) displayed signals for transcriptional activity of LATs (Table 2). All positive and negative control samples gave the expected results. These findings indicate that the EHV DNA detected in the trigeminal ganglia was in the latent state. Latent herpesvirus DNA was not detected in any of the tested lymph nodes. Interestingly, these results together with the PCR results showed that zebra (CP4) was co-infected with both EHV-1 (lytic) and EHV-9 (latent) virus.

### 3.4. Genetic Diversity and Phylogenetic Analyses

The mean nucleotide diversity (π) estimated for EHV-1 and EHV-9 based on *gB* sequences was 0.008% (± 0.004) and 0.004% (± 0.002), respectively. The π value based on *Pol* sequences was estimated at 0.007% (± 0.002) and 0.001% for EHV-1 and EHV-9, respectively. These results indicate lower genetic diversity among EHV-9 sequences compared with EHV-1.

**Figure 3:**
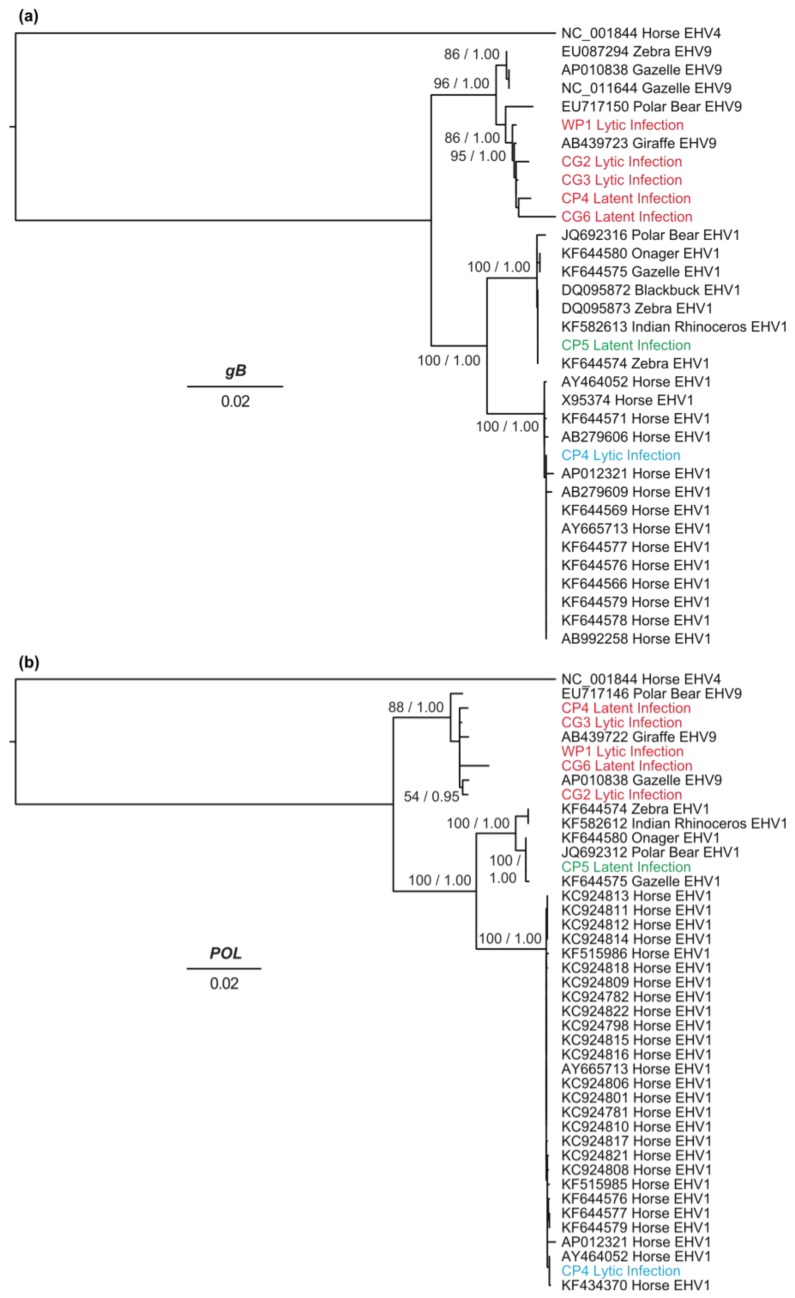
Phylogenetic trees inferred using maximum likelihood from nucleotide sequences of (**a**) *gB* and (**b**) *Pol* genes for the six zebras WP1, CG2, CG3 (Equine herpesvirus 9 (EHV-9) lytic infection), CP4 (Equine herpesvirus 1 (EHV-1) lytic and EHV-9 latent infection), CP5, and CG6 (EHV-1 and EHV-9 latent infection, respectively) and other equine herpesviruses. Reference sequences are indicated by GenBank accession number, species from which the sequence was isolated, and viral strain. The novel EHV-9 sequences are in **red**, the novel EHV-1-horse like zebra sequence is in **blue**, and the novel zebra-EHV-1 sequence is in **green**. The trees are shown with branches lengths scaled to nucleotide substitutions per site. Selected nodes are labeled with maximum-likelihood bootstrap support values and posterior probabilities, separated by a slash “/”.

The phylogenetic relationships of the detected EHV-1 and EHV-9, of lytic and latent infections, were inferred from the sequences of the *Pol* and *gB* genes (Figure 3) as well as the *UL49.5* gene (Figure S2). Virus sequences from lytic infection in WP1, CG2, and CG3, plus the latent infections in CP4 and CG6 exhibited 98%–99% similarity to EHV-9 reference sequences. The *gB* sequence of the detected EHV-9 either in captive or wild zebras clustered with a giraffe EHV-9 isolate, forming a sister clade to gazelle EHV-9 isolates (Figure 3a). The phylogenetic tree inferred from the *Pol* sequences showed that the CG2 lytic, WP1 lytic, CG3 lytic, CP4 latent, and CG6 latent sequences clustered with EHV-9 sequences from gazelle and giraffe (Figure 3b).

The tree inferred from *UL49.5* sequences confirmed that the detected isolates belong to the EHV-9 clade (Figure S2). CP4 latent sequences were not included in the phylogenetic analysis of *UL49.5* due to insufficient DNA. Phylogenetic analyses of EHV-9 viruses circulating in zoos demonstrated minimal differences among sequences from captive animals, with only small divergences from the wild strain. In contrast, the partial coding sequences of the three genes determined from the lytic infection of CP4 clustered with horse EHV-1 sequences. CP5 sequences (latent infection) were placed in a group that includes EHV-1 from gazelle, polar bear, onager, Indian rhinoceros, and zebra, and is more distantly related to horse-derived EHV-1 sequences (Figure 3 and Figure S2).

## 4. Discussion

A key property of alphaherpesviruses is their ability to establish life-long latency in natural hosts where they can evade the host immune system [38]. This makes elimination of herpesviruses almost impossible and helps them to maintain long-term infections in their hosts. Upon stimulation by various cues, the virus can reactivate and be transmitted to susceptible hosts, which may occur with or without overt clinical disease. Uncontrolled reactivation can have a strong impact on zoos, where different susceptible animal species (including endangered animals) are kept together and can result in fatal cross-species transmissions. There have been no previous reports of EHV-1 or EHV-9 latency in zoo animals, except for EHV-1 in horses. Only one study described the possible existence of latent EHV-9 infection in a wild zebra [10]. However, the authors did not confirm the actual state of latency; for example, absent transcriptional activity of the *gB* gene and presence of LATs was not demonstrated. The conclusion of latency was based on the presence of viral *gB* DNA in the tested samples.

In the current study, we confirmed latent EHV-1 and EHV-9 infections in zebras by detection of active LAT transcripts with the simultaneous absence of gB mRNA in the sensory ganglia of zebras. Recently, EHV-1 and EHV-9 have been shown to spread beyond their natural hosts and to infect other zoo animal species, causing fatal neurological disorders [4,5,6,7,24,25]. Latency of EHV-1 and EHV-9 in captive zebras suggests that the threat is determined by reactivation in latent individuals, which is currently not predictable. However, clinically healthy latent animals could reactivate and threaten both natural and unexpected hosts.

None of the three latently infected animals showed clinical signs or lesions related to EHV infection, except for the co-infected animal (CP4) in which EHV-1 was lytically replicating. The animal displayed signs of ataxia and tremors before death. Among the three EHV-9-infected zebras, signs of ataxia followed by nervous manifestations were detected in one zebra. Previous reports only described nervous manifestations associated with EHV-9 infection of unnatural hosts, but not equids [4,6,7]. Even after experimental infection of horses with EHV-9, only viremia was detected without evidence of neuronal manifestations [39]. The ability of the virus to reach the central nervous system (CNS) inducing histopathological changes was detected under both natural and experimental infection of unnatural hosts, with the forebrain predominantly affected [6,40,41,42,43,44]. In the current study, non-suppurative encephalitis was detected for the first time in the forebrain and cerebral cortex in a zebra. However, we could not get unambiguous signals for the virus antigen with immunohistochemistry. At present, it is not clear whether this is because of issues with antibody specificity or the preservation of the tissues. We assume the latter, as the background with the antibodies tested was substantial. Whether EHV-9 reaches the nervous system through the nervous tissue or blood remains to be investigated.

Previous reports have described the contribution of homologous recombination to the evolution of many herpesviruses, including herpes simplex virus 1 (HSV-1) and 2 (HSV-2), EHV-1, EHV-4, and varicella-zoster virus (VZV) [21,22,23,45,46,47]. In general, recombination requires co-infection and simultaneous replication of two or more viral strains in the same cell. However, little is known about the exact mechanism of how recombination occurs and if recombination events happen directly after primary infection, during latency, or during reactivation [21]. Recently, a recombinant EHV-1 containing EHV-9 sequences in the *Pol* gene was detected in a polar bear and Indian rhinoceros, both of which died after nervous manifestations in Germany [24,25]. It was postulated that zebra might be the host where this recombination took place [25]. We demonstrated the co-occurrence of EHV-1 and EHV-9, with high prevalence, among zebras through detection of neutralizing antibodies of both viruses using a discriminatory ELISA assay [8]. In the current study, we have been able to confirm this result further with the detection of co-infection with both EHV-1 and EHV-9 in a captive plains zebra (CP4). Taken together, the results support the notion that the zebra serves as a natural host for both viruses, potentially allowing recombination to take place. In turn, this could be one of several possibilities that could lead to the generation of viral strains with altered host specificity and virulence.

In contrast with apparently common variation generated by recombination among EHVs, little sequence variation was observed for EHV-9 in the current study. This result suggests that the evolution of this virus is slow or that natural selection constrains diversification. However, we observed two clades, one grouping gazelle and zebra sequences, and another grouping the polar bear, giraffe and all of the zebra EHV-9 sequences identified in the current study. EHV-1 demonstrated higher sequence diversity, with a clade predominantly grouping domestic horses and a mixed species clade containing the recombinant polar bear, Asian rhinoceros, onager and zebra sequences. Surprisingly, the EHV-1 sequence of the captive zebra (CP4) exhibiting latent EHV-9 infection and lytic EHV-1 infection was most closely related to that of horse EHV-1. Whether this indicates that the zebra was infected by domestic horses in captivity, or whether zebras carry this strain naturally, remains to be confirmed in further epidemiological studies.

Virus diversification, co-infection and subsequent recombination have the potential to influence the pathogenicity, virulence and host range of different viruses. All of these variables may increase the severity and outcome of disease episodes, which have to take into account the growing list of the alphaherpesviruses detected recently in different animal species. Latency and reactivation constitute an important epidemiological advantage for the virus, given that sporadic cases of abortion and neurological disease can occur without an external source of infection. This, in particular, may have pathogenic consequences in zebras and subsequently in other animals, leading to further fatal cross-species transmissions.

## Figures and Tables

**Table 1:** List of positive animals tested in the study and virus infection status.

Animal Species	Sample ID	Sex	Age (Years)	Observations	Infection
Plains zebra	WP1	f	2	unknown	Lytic EHV-9
Plains zebra	CP4	m	24	Signs of ataxia and tremors without improvement after the course of medication (complete rest and anti-inflammatory drug, Meloxicam)	Lytic EHV-1+ Latent EHV-9
Plains zebra	CP5	m	2	Cardiomyopathy was noticed during necropsy	Latent zebra-borne EHV-1
Grevy’s zebra	CG2	f	23	Found lying in the barn. No clinical signs were observed. Intramuscular bleeding along the femoral shaft was found during necropsy	Lytic EHV-9
Grevy’s zebra	CG3	f	18	Central nervous disorders	Lytic EHV-9
Grevy’s zebra	CG6	m	11	Massive intra-abdominal hemorrhage followed by hypovolemic shock as a result of surgical complications	Latent EHV-9

Equine herpesvirus 1, EHV-1; Equine herpesvirus 9, EHV-9; f, female; m, male.

**Table 2:** Latent viral DNA and RNA copies tested by qPCR in zebra.

Samples ID	Viral Genomic DNA *(gB)*, (copies/million cells)	Transcriptional Activity of *gB* (mRNA)	Transcriptional Activity of LATs (copies/million cells)	Virus Species
CP4	8	Negative	107	EHV-9
CP5	65.3	Negative	124	EHV-1
CG6	33.3	Negative	174	EHV-9

## Supplementary Materials

The following are available online at www.mdpi.com/1999-4915/8/9/262/s1.
