# Supplementary material for: Zebra Alphaherpesviruses (EHV-1 and EHV-9): Genetic Diversity, Latency and Co-Infections
Source: Viruses. 2016 Sep 20;8(9):262. doi: 10.3390/v8090262 (PMC5035975; doi:10.3390/v8090262)
Supplement: Supplementary file 1 [file viruses-08-00262-s001.pdf]

Supplementary Materials: Zebra Alphaherpesviruses (EHV-1 and EHV-9): Genetic Diversity, Latency and Co-Infections

Azza Abdelgawad, Armando Damiani, Simon Y. W. Ho, Günter Strauss, Claudia A. Szentiks, Marion L. East, Nikolaus Osterrieder and Alex D. Greenwood

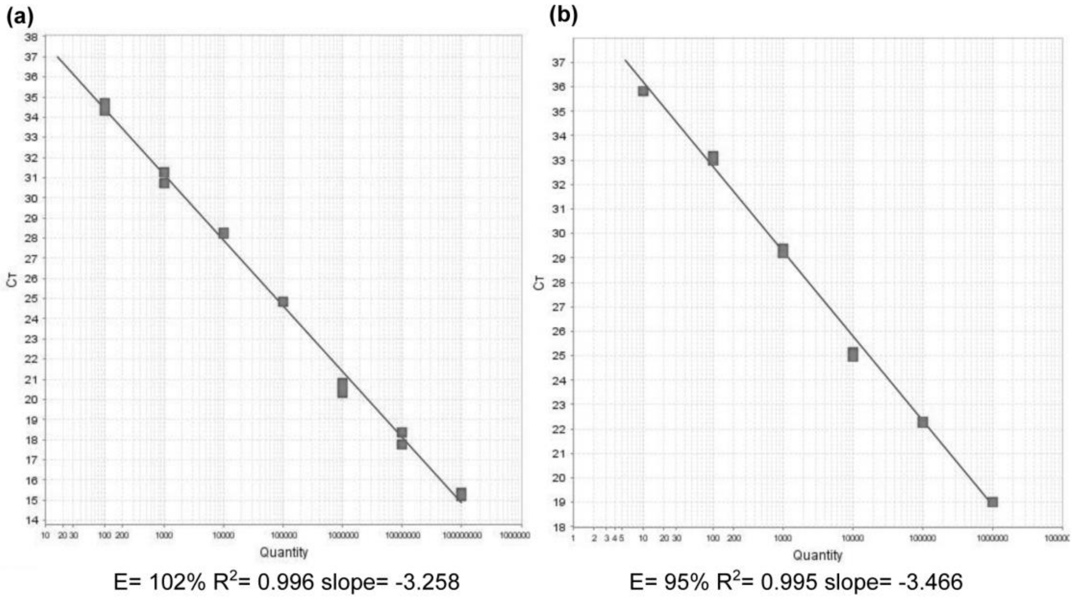

Figure S1. The quantitative real-time PCR (qPCR) standardizations. The standard curve of 10-fold serial dilutions of (**a**) *gB* and (**b**) ORF63 are shown with a correlation coefficient (R2) of 0.996 and 0.995, respectively.

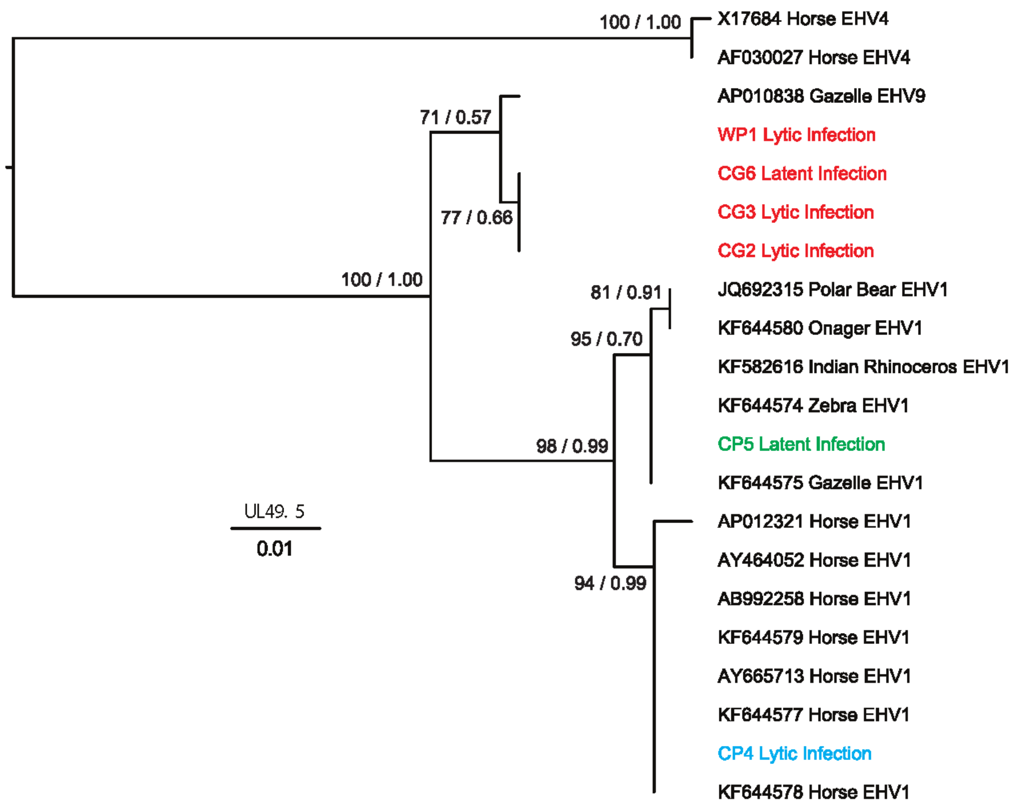

Figure S2. Phylogenetic tree inferred using maximum likelihood from nucleotide sequences of *UL49.5* gene for the six zebras WP1, CG2, CG3 (EHV-9 lytic infection), CP4 (EHV-1 lytic and EHV-9 latent infection), CP5, and CG6 (EHV-1 and EHV-9 latent infection, respectively) and other equine herpesviruses. The novel EHV-9 sequences are in **red**, the novel EHV-1-horse like zebra sequence is in **blue**, and the novel zebra-borne EHV-1 sequence is in **green**. Selected nodes are labeled with maximum likelihood bootstrap support values and posterior probabilities, separated by a slash "/".

Table S1. List of equid animals analyzed in the study.

Species/subspecies	Origin	Number of Tested Animals
Equus quagga	Wild plains zebra (WP)	7 *
Equus quagga boehmi	Captive plains zebra (CP)	3; 1 *
Equus grevyi	Captive Grevy's zebra (CG)	3
Equus zebra hartmannae	Captive Hartmann's mountain zebra	2
Equus africanus somalicus	Captive Somali wild ass	6; 3 *
Equus africanus asinus	Captive donkey	3 *

* Ganglia and lymph nodes were not collected from these animals.

Table S2. List of new primers and probes used in the study.

Primer	Sequence
Pol	
AZ11 (F)	5′-AATGTGCGATCTCAGCTTTG-3′
AZ14 (R)	5′-GATCTTTTGTTGTACGACGA-3′
AZ1 (F)	5′-TACAACAAAAGATCTACCAG-3′
AZ2 (R)	5′-GATAGCCAAAGCCACGCCTT-3′
AZ12 (F)	5′-CGTGGCTTTGGCTATCCATA-3′
AZ15 (R)	5′-ATCTCCTGTCTGCTGTACTC-3′
AZ9 (F)	5′-AGGTCCTCTTGGTTAGTTGC-3′

AZ17 (R)	5′-TTAAATTTACACAGACATG-3′
gB	
gB1 (F)	5′-CCATGTCAACGCACTCCC-3′
gB1 (R)	5′-ACAATATCACCGGTGGACAG-3′
gB2 (F)	5′-CTGTCCACCGGTGATATTGT-3′
gB2b (R)	5′-GGTACGGACAGGAGAGACCT-3′
gB (F)	5′-CTTGTGAGATCTAACCGCAC-3′
gB (R)	5′-GGGTATAGAGCTTTCATGGGG-3′
gB3 (F)	5′-AGATATGTAATGCAGATCCG-3′
gB3b (R)	5′-AAATATGAGGTCACACTTT-3′
ORF69–ORF74	
US3 (F)	5′-GACCACCTAACCGACTGGTT-3′
US3 (R)	5′-CGCGTGTAGGGCTTGCGCTC-3′
US4 (F)	5′-CTACCCCTGCTTTCAACGCG-3′
US4 (R)	5′-TGTGTGACTCCCACGAGTGA-3′
US5 (F)	5′-CTTACCCAAATACGCTGAGG-3′
US6 (R)	5′-TCTCGTATGTTGACGAGCCCA-3′
US6 (F)	5′-GCCGCTACAACCACAGCTGT-3′
US7 (R)	5′-AAGCGAAGTTGGAAGTTGAG-3′
US8 (F)	5′-TTAGTGGCTGCGACCACGCT-3′
US8 (R)	5′-ATCCGGAGGCACGGGTCTTG-3′
US9 (F)	5′-CCGGATAACCACCCTGGATT-3′
US10 (R)	5′-CCCCACGCATCGAGTACTGT-3′
US11 (F)	5′-AGTCCAACAAGTTGAACTTT-3′
US11 (R)	5′-TTCATAAAGTGATTTGCGGT-3′
LAT-specific primer	5′-CTGGCTGGTCGAAAGGCTCG-3′
qPCR	
ORF63_LAT (F)	5′-GTGTCTTCGTGAAACATCGG-3′
ORF63_LAT (R)	5′-TGCGGAACATTGTTATGGAT-3′
ORF63_LAT (probe)	5′FAM-TCCTCGTTACAGCCATGCTCGC-TAMRA3′
ORF63 (oligo)	5′GATGAGATCCGTGACAAAGGGCACAGTGTCTTCGTGAAACATCGGCCAAAACTGGCGAGTGAGCTCTTCCTCGTTACAGCCATGCTCGCACAGTGTATCCATAACAATGTTCCGCATCAC-3′
B2M (F)	5′-ATG GAAAGCCAAATTTCCTG-3′
B2M (R)	5′-ACCGGTCGACTTTCATCTTC-3′
B2M (probe)	5′HEX-TGGGTTCCATCCGCCTGAGA-BHQ13′
B2M (oligo)	5′-AGAGAATGGAAAGCCAAATTTCCTGAACTGCTATGTCTCTGGGTTCCATCCGCCTGAGATTGAAATTGATTTGCTAAAGAATGGAGAGAAGATGAAAGTCGACCGGTCAGAC-3′
